# Alcohol consumption and the use of marijuana in young adults

**DOI:** 10.1186/1940-0640-7-S1-A39

**Published:** 2012-10-09

**Authors:** Ladislav Csemy, Hana Sovinova, Bohumir Prochazka

**Affiliations:** 1 National Institute of Public Health, Prague, Czech Republic

## 

The main objective of this study was to explore associations between alcohol consumption and marijuana use in young adults and to discuss opportunities for brief intervention (BI). Face-to-face structured interviews were carried out with 2221 young adult Czechs (mean age, 29.9; SD, 5.8 years). Of the total sample, 51.4% were males. Alcohol consumption was calculated using a beverage-specific quantity/frequency method. Alcohol-related problems were assessed using the Czech version of the Alcohol Use Disorders Identification Test (AUDIT). Participants were also asked about the frequency of marijuana use in the last 12 months. Overall past-year alcohol consumption per individual was 9.2 L of ethanol. The past-year prevalence of marijuana use was 21.8%. The use of marijuana positively correlated with the frequency of beer drinking (r = 0.27), frequency of heavy episodic drinking [HED], (r = 0.32) and with the AUDI summary score (r = 0.39). People with harmful or problem drinking (AUDIT score > 16) reported marijuana use more frequently than people who drank moderately (60% compared with 18.8%; odds ratio [OR] = 6.54; 95% CI = 4.7; 9.1). The OR for marijuana use in people with HED was 4.3 (CI = 3.3; 5.6). These results suggest that frequent HED and harmful drinking are closely associated with marijuana use in younger adults. As marijuana use (including heavy use) is common in the Czech Republic, extending screening and BI to reduce the use of cannabis may be recommended. The existing guidelines for BI should be modified to cover marijuana consumption as well.

